# Integrins and Epithelial-Mesenchymal Cooperation in the Tumor Microenvironment of Muscle-Invasive Lethal Cancers

**DOI:** 10.3389/fcell.2022.837585

**Published:** 2022-03-01

**Authors:** William L. Harryman, Kendra D. Marr, Ray B. Nagle, Anne E. Cress

**Affiliations:** ^1^ Cancer Biology Graduate Interdisciplinary Program, University of Arizona Cancer Center, Tucson, AZ, United States; ^2^ Cancer Biology Graduate Interdisciplinary Program, University of Arizona, Tucson, AZ, United States; ^3^ Medical Scientist Training Program, College of Medicine, University of Arizona, Tucson, AZ, United States; ^4^ Department of Pathology, College of Medicine, University of Arizona, Tucson, AZ, United States; ^5^ Department of Cellular and Molecular Medicine and Department of Radiation Oncology, College of Medicine, University of Arizona, Tucson, AZ, United States

**Keywords:** integrin, growth factor, muscle invasion, cadherin, epithelial mesenchymal cooperation

## Abstract

Muscle-invasive lethal carcinomas traverse into and through this specialized biophysical and growth factor enriched microenvironment. We will highlight cancers that originate in organs surrounded by smooth muscle, which presents a barrier to dissemination, including prostate, bladder, esophageal, gastric, and colorectal cancers. We propose that the heterogeneity of cell-cell and cell-ECM adhesion receptors is an important driver of aggressive tumor networks with functional consequences for progression. Phenotype heterogeneity of the tumor provides a biophysical advantage for tumor network invasion through the tensile muscle and survival of the tumor network. We hypothesize that a functional epithelial-mesenchymal cooperation (EMC)exists within the tumor invasive network to facilitate tumor escape from the primary organ, invasion and traversing of muscle, and navigation to metastatic sites. Cooperation between specific epithelial cells within the tumor and stromal (mesenchymal) cells interacting with the tumor is illustrated using the examples of laminin-binding adhesion molecules—especially integrins—and their response to growth and inflammatory factors in the tumor microenvironment. The cooperation between cell-cell (E-cadherin, CDH1) and cell-ECM (α6 integrin, CD49f) expression and growth factor receptors is highlighted within poorly differentiated human tumors associated with aggressive disease. Cancer-associated fibroblasts are examined for their role in the tumor microenvironment in generating and organizing various growth factors. Cellular structural proteins are potential utility markers for future spatial profiling studies. We also examine the special characteristics of the smooth muscle microenvironment and how invasion by a primary tumor can alter this environment and contribute to tumor escape via cooperation between epithelial and stromal cells. This cooperative state allows the heterogenous tumor clusters to be shaped by various growth factors, co-opt or evade immune system response, adapt from hypoxic to normoxic conditions, adjust to varying energy sources, and survive radiation and chemotherapeutic interventions. Understanding the epithelial-mesenchymal cooperation in early tumor invasive networks holds potential for both identifying early biomarkers of the aggressive transition and identification of novel agents to prevent the epithelial-mesenchymal cooperation phenotype. Epithelial-mesenchymal cooperation is likely to unveil new tumor subtypes to aid in selection of appropriate therapeutic strategies.

## 1 Introduction

Several smooth muscle invasive cancers, including prostate, bladder, esophageal, gastric, and colorectal, rank in the top 10 cancers for incidence and deaths (Society AC, 2020). These cancers share a common feature—when organ confined, they are not lethal with treatment; they become lethal when they escape the primary organ and metastasize. A primary escape route for these tumors from the original site is invasion through the smooth muscle surrounding the exterior of the organ (Beunk et al., 2019) to intravasate or extravasate through vessels, which requires tumor clusters to migrate through a smooth muscle layer sheathing the vein wall, and move through an even thicker layer in escaping through arteries (Miller et al., 2009).

The epithelial to mesenchymal transition (EMT), a major event in tumor metastasis (Birchmeier and Birchmeier, 1995; Hay and Zuk, 1995; Birchmeier et al., 1996), although not without some dissent (Tarin et al., 2005), is defined as epithelial plasticity typified by enduring “morphological and molecular changes” in epithelial cells—the result is a phenotypic switch by which epithelial cells acquire a mesenchymal cell type (Van Marck). The EMT hypothesis also requires that the cells reverse their phenotypic switch (mesenchymal to epithelial transition, MET) to enter the metastatic site (Kalluri and Weinberg, 2009). The evolution of the epithelial to mesenchymal transition phenotype hypothesis includes the notion that the loss or gain of an epithelial phenotype in carcinoma progression is a binary switch (EMT or MET), a spectrum of expression within a tumor, a partial or hybrid state, or the result of epithelial-mesenchymal plasticity (EMP) (Williams et al., 2019; Bakir et al., 2020; Sinha et al., 2020). The transitions are indicative of an unstable cancer phenotype referred to as phenotypic plasticity. EMT and MET phenotypes can be modulated by both genetic and epigenetic mechanisms (reviewed in Pastushenko and Blanpain (2019)) and in response to an ever-changing tumor microenvironment (reviewed in Dongre and Weinberg (2019)). The majority of EMT research investigates the activities of single cells.

Successful metastasis most likely occurs, however, because of tumor clusters, not individual circulating tumor cells (CTCs), in a variety of cancers, including prostate, oral squamous cell carcinoma, colorectal carcinoma, melanoma, and breast cancer (Friedl and Gilmour, 2009; Aceto et al., 2015; Harryman et al., 2016). Tumor clusters display a hybrid epithelial/mesenchymal (E/M) phenotype in which some cells exhibit epithelial traits of cell-cell adhesion and other cells exhibit mesenchymal characteristics of migration and invasion (Jolly et al., 2018). The analysis of cell mixtures within model systems suggests that leader and follower cells have distinct phenotypes to move a tumor mass as a collective of cells (Friedl and Gilmour, 2009). Futhermore, Friedl highlights the role of the actin cytoskeleton in generating traction and protrusion force for migration and in maintaining cell-cell junctions in cell clusters, a process mediated by distinct receptor-ligand pairs that vary based on context (Friedl and Gilmour, 2009).

One limitation in understanding the regulation of loss or gain of an epithelial phenotype is that within a single patient tissue sample, a heterogeneity of phenotype is often observed, reported as a mixed phenotype or aberrant expression. Examples of tumor heterogeneity include the presence of E-cadherin (cell-cell adhesion) and integrin (cell-ECM adhesion) expression within different populations of tumor in prostate, bladder, esophagus, ovaries, and the colon. The heterogeneity of tumor cell-type expression within the same patient is more often the rule rather than is a loss of the epithelial phenotype during progression (Ahmed and Gravel, 2018). The heterogeneity of prostate tumors, in particular, is revealed in multiple epithelial cell types, mixed with stromal cells, endothelial cells, macrophages, lymphocytes, and cancer associated fibroblasts (CAFS, also known as carcinoma associated fibroblasts), with all of these cells interacting as a complex adaptive system (Schwab and Pienta, 1996) to determine tumor progression and treatment outcomes that are unique to each patient (Maitland et al., 2019).

A second limitation is that comparisons are often made between mRNA expression levels within normal glands as compared to those in cancer, whereas the abundance of only approximately 50% of gene products are accurately reflected by their mRNA expression levels (Labrie et al., 2019). A third limitation is that if protein levels are assessed, the normal function of the gene product in the context of a tumor is assumed, which may or may not be warranted. Examples are provided indicating different roles in cancer as compared to normal tissue that accompany the altered expression patterns of cell-cell and cell-ECM adhesion receptors.

We propose that the heterogeneity of cell-cell and cell-ECM adhesion receptors is an important driver of aggressive tumor networks and has functional consequences in cases of muscle invasive and lethal carcinomas. The heterogeneity is functional in that it provides a biophysical advantage for invasion of tumor networks through the tensile muscle and survival of the tumor network during progression (Harryman et al., 2019). Genomic heterogeneity also arises in human prostate tumors within the muscle rich organ as multiple spatially intermixed lineages that persist within distant lesions (Woodcock et al., 2020). Similarly, genomic imbalances are widespread in muscle invasive bladder cancers, more frequently than lower grade/stage tumors (Spasova et al., 2021). Understanding the genomic heterogeneity of muscle invasive bladder cancer is now being explored as a potential therapeutic response tool (Lotan et al., 2021), an approach amenable to understanding the consequences of adhesion receptor phenotype heterogeneity.

## 2 Cell-Cell (E-Cadherin, CDH1) and Cell-ECM (α6 Integrin, CD49f) Expression Within Poorly Differentiated Human Tumors Associated With Aggressive Disease

One protein structural marker for EMT is vimentin, an intermediate filament protein associated with cellular motility, cell shape maintenance, and directional migration of cells (Ivaska, 2011; Maehira et al., 2019). Another marker, Epithelial-cadherin (E-cadherin), a cell-surface adhesion molecule, is essential in maintaining apical-basal polarity in epithelial cells (cell-cell adhesion), and is known to decrease during EMT, while also known to be present in metastases (Petrova et al., 2016), suggesting metastasis may not be solely dependent on early EMT, but more likely a result of a hybrid or E-M cooperative phenotype in migrating tumor clusters (Jolly et al., 2019; Sinha et al., 2020). In the following examples, reports of loss, gain and heterogeneity of E-cadherin protein expression in aggressive tumor types are shown to point out the current state of our understanding.

Zhou et al., found that patients whose primary tumors express high vimentin levels with loss of E-cadherin expression were more likely to experience lymph node metastasis, distant metastasis, perineural invasion, and advanced staging (American Joint Committee on Cancer stage) than patients with high or low vimentin expression and preserved E-cadherin expression or low vimentin expression and loss of E-cadherin expression (Zhou et al., 2021). As previously reported (Maehira et al., 2019), increased vimentin expression and loss of E-cadherin expression in the cooperative phenotype of tumor clusters results in metastasis, invasion, radio-resistance, and generation of cancer cells with stem cell-like characteristics in pancreatic cancer. Loss of E-cadherin, and the transition to a more aggressive phenotype, requires the coordinated regulation of both cell-cell (E-cadherin-mediated) adhesions and cell-ECM (integrin-mediated) adhesions, both processes being regulated by a variety of factors within the tumor microenvironment (Canel et al., 2013).

Gao et al., also found that ultrasound-guided needle biopsies of pancreatic adenocarcinoma revealed decreasing intensity of membranous staining of E-cadherin in poorly differentiated specimens (Gao et al., 2013). However, this was not universal, as many poorly differentiated samples still exhibited considerable E-cadherin expression (14 of 27 poorly differentiated samples showed moderately to strongly positive E-cadherin expression), suggesting that cell-cell adhesions (cohesive tumor cell clusters) were likely still present (a mixed E-M cooperative phenotype).

Zhao et al., found that in esophageal cancers, when looking at early stage (I, II) tumors, decreased or lost E-cadherin expression resulted in significantly shorter survival (both overall and disease-free) (Zhao et al., 2003). Patients with grade III or IV tumors exhibited shorter survival timeframes regardless of E-cadherin expression. However, approximately 15% of poorly differentiated tumors (highly aggressive) contained normal levels of E-cadherin (Zhao et al., 2003).

In a unique form of gastric cancer—hereditary diffuse gastric cancer—Figueiredo et al., found that β1 integrin regulates invasion in E-cadherin dysregulated tumors (Figueiredo et al., 2022). Identifying three E-cadherin variants associated with aggressive gastric cancer (A634V, R749W, and V832M), the authors concluded that dysfunctional E-cadherin variants activate a specific mechanotransduction program to regulate matrix adhesion (Figueiredo et al., 2022). Having determined that Fibronectin + Vitronectin offered an ECM environment conducive to the adherence of the mutant E-cad cells, they found significantly higher traction forces on Fibronectin + Vitronectin gels when compared to the wild-type E-cad cells (Figueiredo et al., 2022). Further, the authors found cells expressing mutant forms of E-cadherin display significant increased levels of β1 integrin when compared to those of the wild-type—additionally, they found a slight increase in β1 integrin (which formed tighter networks than when β4 integrin was present) when β4 integrin was inhibited (Figueiredo et al., 2022). Finally, the authors found that E-cadherin loss and a corresponding increase in β1 integrin expression is associated with higher grade tumors and reduced overall patient survival (Figueiredo et al., 2022).

Similarly, in bladder cancers, heterogenous E-cadherin staining was found, with E-cadherin expression occurring both at the plasma membrane of the surface epithelium of the normal urinary bladder and at the cell-cell boundaries of some tumors (Fujisawa et al., 1996). In the same study, tumor grade was associated with decreased expression of E-cadherin. Using their cutoff of 50% or more abnormal cells, 25% of grade I, 46% of grade II, and 86% of grade III specimens showed decreased staining for E-cadherin. However, a more recent study found that in muscle-invasive bladder cancer (MIBC), abnormal cadherin patterns (decreased E-cadherin and increased N-cadherin) were highest (70.3% of cases) and associated with adverse prognostic indicators (Shash et al., 2021). More interestingly, 35% of the samples in this study showed a hybrid pattern of cadherin expression (both E- and N-cadherin positive) (Shash et al., 2021), suggesting the possibility of the E-M cooperation we hypothesize.

Both primary colorectal cancer (CRC) and corresponding metastatic lymph nodes displayed a predominantly membranous expression (pure or mixed) of E-cadherin (67.69% and 89.23%, respectively) (Palaghia et al., 2016). Weiss et al. (2011), found increased levels of soluble E-cadherin, an 80 kDa protein fragment resulting from the proteolytic cleavage of the extracellular domain of full-length E-cadherin (Repetto et al., 2014), in patients with advanced CRC (stages III and IV). Okugawa et al., found that elevated levels of soluble E-cadherin in preoperative CRC were associated with poor prognosis (Okugawa et al., 2012). Many cancer studies suggest that decreased E-cadherin is associated with aggressive disease and/or poor outcomes, while others, such as these, suggest the opposite. Taken together, the studies indicate that aberrant E-cadherin levels, distribution, and perhaps function occurs during tumor progression. Dissemination through the lymph nodes or through the vascular system are different microenvironments where tumors utilize a cohesive adhesion phenotype (reviewed in Harryman et al. (2019), Harryman et al. (2021).

Another example of a normal cell adhesion receptor, α6β4 integrin, required for a strong adhesion structure, known as the hemidesmosome (Te Molder et al., 2021), is aberrantly used in cancer progression. Loss of the hemidesmosome structure dramatically alters the context of the tissue resulting in early cancers (De Arcangelis et al., 2017; Wang et al., 2017). Along with the decrease in E-cadherin expression in CRC noted above, an overexpression of both α6 and β4 integrin subunits occurs in the primary tumor as well as in established CRC cell lines, with the relocalization of α6β4 to the actin cytoskeleton, resulting in a more migratory and anoikis-resistant phenotype (Beaulieu, 2019). The role of α6β4 integrin in tumor progression has been observed and studied in skin, breast, and colon cancers [reviewed (Ramovs et al., 2017)]. In prostate cancer, α6β4 integrin expression is lost in early cancer progression and is replaced by the α6β1 heterodimer expression, whereas α6β4 expression persists in colon and breast cancer (Davis et al., 2001). Haraguchi et al., identified integrin α6 as an important marker for detecting colorectal cancer stem cells (CSCs), which possess the ability to self-renew, have high tumorigenic activity, show resistance to anticancer drugs and radiation, and cause cancer recurrence (Haraguchi et al., 2013). Taken together, the studies indicate that the α6 integrin either paired with the β4 or β1 subunit, is aberrantly expressed and used during tumor progression.

Strauss et al., identified subpopulations of ovarian cancer cells that are in a transitory E/M hybrid stage, i.e., cells that express both epithelial and mesenchymal markers (Strauss et al., 2011). Further, they determined that primary ovarian cancer cultures are phenotypically heterogenous and that tumor-initiating cells display attributes of mesenchymal and epithelial (E/M) cells. However, they also identified phenotypic subsets based, in part, on subcellular localization of E-cadherin—*in vivo* xenograft tumor growth in SCID nude mice was driven by E/M-MP cells (membrane E-cadherin^low^/cytoplasmic E-cadherin^high^/CD133^high^, CD44^high^, Tie2^low^), which produced epithelial ovarian cancer cells (Strauss et al., 2011). Under *in vitro* conditions, these same E/M-MP cells differentiated into mesenchymal cells, replicating traditional pathways associated with an epithelial-to-mesenchymal transition (Strauss et al., 2011). This data suggests that EMT models based on *in vitro* observations may be inaccurate. In another study involving ovarian cancer, Wei et al., found that expression of α6 integrin was associated with multi-drug resistance and poor prognosis in ovarian cancer (Wei et al., 2019). Similarly, Dhaliwal and Shepherd review the role of integrin-mediated cell adhesion in epithelial ovarian cancer progression and report that laminin interaction with α6β1 integrins participates in mediating ovarian tumor spheroid formation (Dhaliwal and Shepherd, 2021).

In prostate cancer, epithelial-mesenchymal transition markers β-catenin, Snail, and E-cadherin do not predict disease free survival (Ipekci et al., 2015); high-grade prostate cancer has reduced E-cadherin expression and some tumors with histologically similar appearance could be distinguished by the presence of mixed populations of E-cadherin negative and E-cadherin positive cells (Umbas et al., 1992). Other researchers have shown paradoxical expression of E-cadherin in prostatic bone metastasis—with some containing mixed patterns of expression (Bryden et al., 1999). Using high-density tissue microarray, 1,220 prostate cancers were analyzed, and 82–90% of cancers were expressing high (normal) E-cadherin. Notably in this study, E-cadherin expression was considered aberrant if less than 70% of the cells had strong membranous staining (Rubin et al., 2001). As discussed, in many epithelial cancers E-cadherin expression decreases (a hallmark of EMT, or the proposed epithelial-mesenchymal cooperation), and there is a corresponding increase of N-cadherin expression (Tran et al., 1999a; Loh et al., 2019). In several prostate cancer cell lines (PC-3N, JCA1, and a subpopulation of PC-3) the increased N-cadherin expression was localized in sites of cell-cell adhesive contacts (Tran et al., 1999a), possibly acting to replace the lost E-cadherin in maintaining cohesive tumor clusters.

**FIGURE 1:**
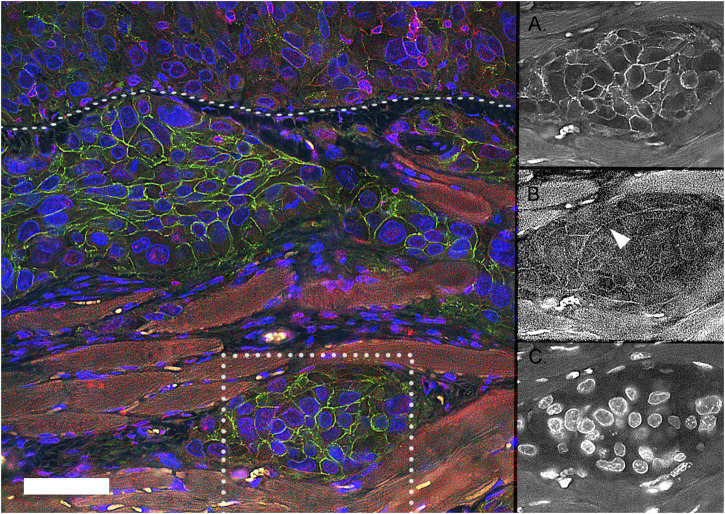
Expression of E-cadherin and α6 integrin in muscle invasive tumor networks in mouse xenograft model. DU145 human tumor cells were IP Injected into a SCID mouse. The resulting human tumor grows on top of and invades through the muscle diaphragm surface (white dotted line). The tumor clusters express E-cadherin (Green), α6 integrin (Red) in spatially distinct cell-cell and cell-ECM regions, with no detectable overlap (Yellow). Blue = DAPI; Green = E-cadherin; Red = ITGA6 (NT); the auto-fluorescent muscle is a dull red. The individual image channels of the tumor splitting the muscle (in the dotted white box) are separated to show the distinct distribution of E-cadherin **(A)**, α6 integrin **(B)**, and nuclei **(C)**. Scale bar = 50 um.

One explanation for the paradoxical reports of aberrant E-cadherin expression may be related to the influence of the tumor microenvironment. For example, in a mouse xenograft model system, our research group has observed a dramatic alteration in E-cadherin expression as tumors move into and through a biophysically challenging smooth muscle. Figure 1 shows the distribution of E-cadherin (green) increases as the tumor breeches a smooth muscle barrier (white dotted line). Tumor clusters that are moving between the muscle layers (Figure 1A) express E-cadherin within the central region of the cluster, with α6 integrin observed in a distinct cell-ECM distribution (Figure 1B) with no detectable overlap with E-cadherin. This observation is consistent with previous work that aggressive prostate tumors within metastatic bone sites express E-cadherin (De Marzo et al., 1999).

Nearly all epithelial cancers involve lost, reduced, or mixed expression of E-cadherin, and with that an assumed loss of cell-cell adhesion; however, it is also true that cohesive clusters of tumor cells are the greater source of metastases (Friedl and Gilmour, 2009; Aceto et al., 2015; Cheung and Ewald, 2016; Fang and Kang, 2021), as individual cells do not survive to seed distant sites (Harryman et al., 2016). Under conditions of low E-cadherin expression, N-cadherin and/or integrins assume the role of cell-cell adhesion indirectly, for example, α5β1 integrin binds to intercellular deposits of fibronectin or α6β1 integrin binds to intercellular laminin (Friedl and Gilmour, 2009). Considering this, the idea of “partial EMT” may be incorrect, as Lu and Lu point out (Lu and Lu, 2021), as it appears that migrating collectives are a “mosaic and heterogeneous population”—referring to this process as “partial EMT” prevents a more adequate understanding of these mechanisms (Lu and Lu, 2021). We therefore propose epithelial-mesenchymal cooperation (EMC) as a more accurate description of the process.

If classical EMT was universal, resulting in single circulating tumor cell (CTC) migration, cohesive clusters of tumor cells would not increase metastasis, but the research shows that clusters present significantly worse clinical outcomes than single CTCs (Aceto et al., 2014; Cheung and Ewald, 2016). These clusters are clearly able to maintain their epithelial characteristics and the corresponding cell-cell adhesion, and in fact, they often seem to maintain E-cadherin expression while single CTCs do not (Aiello et al., 2018). Supporting this, Gloushankova et al., determined that a hybrid epithelial-mesenchymal phenotype (a cooperative phenotype) requires the presence of E-cadherin for cancer cell dissemination, buttressing our hypothesis that E-cadherin and E-cadherin-based adherens junctions (AJs) are necessary for collective invasion and tumor migration (Gloushankova et al., 2017).

### 3 MUSCLE AS A SPECIALIZED TUMOR MICROENVIRONMENT

#### 3.1 The Tumor Microenvironment, ECM, Cancer-Associated Fibroblasts, and Integrins

Inter/intracellular communication in the tumor microenvironment (TME) is driven by a complex and adaptive network of cytokines, chemokines, growth factors, inflammatory, and matrix transforming enzymes all existing in tissue that presents with major disruptions to its physical and chemical properties (Balkwill et al., 2012). Among the specific factors identified by Balkwill et al., are T cells, B cells, natural killer (NK) cells and natural killer T (NKT) cells, tumor-associated macrophages (TAMs), myeloid-derived suppressor cells (MDSCs), cancer-associated fibroblasts (CAFs), adipocytes, and pericytes (perivascular stromal cells) (Balkwill et al., 2012). The ECM provides a foundation for all the cells in the TME, so it plays a central role in the development and metastasis of tumors, especially as the adhesion of a cell to the ECM is key to its movement out of and into the TME (Balkwill et al., 2012). The ECM also contains proteins contributing to angiogenesis, chemokines that stimulate cell migration, and other key growth factors that interact with cell surface receptors and give each tissue its rigidity and elasticity (Balkwill et al., 2012). Tumors are typically stiffer than the surrounding normal tissues due to an increased ECM deposition by cancer-associated fibroblasts that express alpha-smooth muscle actin (α-SMA) (Bauer et al., 2020), allowing the tumor to biomechanically interact with and respond to the stiffness of the ECM (Wullkopf et al., 2018).

Gkretsi and Stylianopoulos offered an excellent review on how cell adhesion and matrix stiffness regulate tumor invasiveness and metastasis (Gkretsi et al., 2018), part of which is summarized here. During tumor progression, the TME is subject to the upregulation of ECM remodeling molecules, such as TGF-β, which has been linked to the development of desmoplasia in tumors (Papageorgis and Stylianopoulos, 2015). Desmoplasia represents a stiffening of the ECM, an “intense fibrotic response,” identifiable by the increase in fibrillar collagen, fibronectin, proteoglycans, and tenascin C collected within the tumor. Desmoplasia is further identified by the increase in inflammatory and tumor-promoting growth factors, including a large population of stromal cells; concurrently, considerable numbers of tissue fibroblasts transition to CAFs containing α-SMA. The increase in CAFs is thought to promote increased numbers of ECM fibers, triggering a parallel increase in desmoplasia. Desmoplasia is associated with ECM stiffening, which leads to negative outcomes in several types of cancer. Integrins allow cells to sense and respond to ECM stiffening via cytoskeletal filaments capable of orchestrating changes within the cell and cell migration. The increased stiffness of the ECM promotes fibronectin production, which binds extracellular collagen, fibrin, and heparan sulfate proteoglycans from one side of the ECM to integrins on the other side. Stiffening of the ECM promotes cell-ECM adhesions that bind the ECM to the cytoskeleton via local adhesion proteins. Integrin clustering stimulates the recruitment of several focal adhesion proteins—including FAK, ILK (which appears to down-regulate E-cadherin (Canel et al., 2013) and regulate integrin expression via kindlin-2 (Kadry et al., 2018)), Src, Rho, and Ras—that promote tumor progression. Taken as a whole, increased ECM stiffness is associated with reduced E-cadherin expression, increased integrin clustering, CAF production, and reduced long-term survival (Gkretsi et al., 2018).

Cancer-associated fibroblasts (CAFs) are large, spindle-shaped mesenchymal cells that share characteristics with smooth muscle cells and fibroblasts, including the expression of both vimentin and α-SMA (Izumi et al., 2016). CAFs are highly heterogeneous and originate from several cell types in the tumor stroma, including fibroblasts, epithelial and endothelial cells (via epithelial/endothelial–mesenchymal transition, EMT/EndMT), bone marrow-derived mesenchymal stem cells, adipose tissue-derived stem cells, pericytes, smooth muscle cells, and vascular smooth muscle cells (Cirri and Chiarugi, 2011; Ghajar et al., 2015; Jang et al., 2019). The heterogeneity of CAFs is also dependent on their site of origin, with differing mechanisms of how CAFs promote tumor progression are likely to differ based on the origin organ (Ghajar et al., 2015). Interestingly, CAFs seem to be activated not only by tumor-derived growth factors and integrins (Jang et al., 2019), but also by mechanical forces within the TME, including hypoxia matrix stiffness (reviewed in Santi et al. (2018)).

CAFs demonstrate powerful tumor-modulating effects by increasing drug resistance (Nurmik et al., 2020; Yoshida, 2020), promoting tumor progression (Yoshida, 2020), the formation of stem cell niches, and immunosuppression (Zeltz et al., 2020), although it must be noted that CAFs also may have anti-tumor effects (Santi et al., 2018; Liu et al., 2019). Immune factors, including cytokines (such as interleukin-11 (IL-11), and interleukin-6 (IL-6)) and chemokines (including IL-6, CCL5, and CXCL10), play a role in converting normal fibroblasts into CAFs, and some of these immune factors act in a feedback loop between the CAFs and the tumor (Yoshida, 2020). For example, CXCL12 secreted from CAFs promotes gastric cancer invasiveness by increasing the clustering of integrin β1 in gastric cancer cells and may lead to tumor progression via increased focal adhesion kinase (FAK) signaling (Izumi et al., 2016; Longmate and DiPersio, 2017). CXCL12 is also known as stromal cell-derived factor 1 (SDF1), a crucial regulator in cancer initiation, angiogenesis, and metastasis (Liu et al., 2019). Moreover, many of these often pro-inflammatory cytokines are secreted by both the tumor cells and the CAFs, and they facilitate the epigenetic modification of CAFs (Yoshida, 2020). CAFs also appear to be involved in altered expression of growth factors such as platelet-derived growth factor (PDGF), insulin-like growth factors I and II (IGF-I,II) (Long et al., 2019), cellular communication network 1 (CCN1, Cyr61 gene) (Joshi et al., 2021), transforming growth factor-β1 (TGF-β1), hepatocyte growth factor/epithelial scatter factor (HGF/SF), and keratinocyte growth factor (KGF) [reviewed in (Olumi et al., 1999)]. In bladder cancer, kindlin-2 knockdown is associated with reduced CAF activation and decreased expression of α-SMA and fibronectin; reduced kindlin-2 also decreased CAF-induced tumor migration and invasion (Wu et al., 2017). Kindlin-2 promotes CAF production in bladder cancer via regulation of TGF-β receptor 1 (TFβ1) and the corresponding downstream induction of the epithelial-mesenchymal cooperative phenotype (Wu et al., 2017; Wang et al., 2020).

CAFs can remodel the ECM to generate pathways for collective cancer cell migration (Gaggioli et al., 2007; Labernadie et al., 2017), although the mechanisms of how cancer cells use CAF-generated tracks and migrate along them are unclear (Labernadie et al., 2017). One theory is that cadherins can enable cells to retain adhesion while controlling front/rear polarization of the leading cells, and Labernadie et al., found that co-cultured CAFs and cancer cells demonstrated co-localization of E-cadherin and N-cadherin at contacts between the two cell types (Labernadie et al., 2017). The presence of E-cadherin would suggest the absence of EMT, however it has been found that carcinoma cells can invade without undergoing traditional EMT, and the tumor cells do not increase mesenchymal markers but do retain cell to cell contact during their invasion—these cells use the mesenchymal characteristics of the CAFs (stromal fibroblasts) to remodel the ECM and consequently follow behind the invading fibroblasts (Gaggioli, 2008).

Stiffness of the ECM, often through mechanotransduction, elicits a wide range of responses from different types of cells; for example, ECM stiffness is central to the differentiation of stromal fibroblasts into CAFs (Emon et al., 2018). Increased ECM stiffness is associated with upregulated expression of α-SMA, a proven myofibroblast (another term for CAFs) marker (Emon et al., 2018). Myofibroblasts are essential in wound healing, and they may arise from a variety of different cell types, including local fibroblasts, pericytes, smooth muscle cells, epithelial cells, endothelial cells, hepatic perisinusoidal cells, mesenchymal stem cells, and fibrocytes (Bagalad et al., 2017). A subset of CAFs act as wound-like myofibroblasts that mediate a disordered chronic wound healing process in tumors; for example, many CAFs share similar features with α-SMA-positive (α-SMA+) myofibroblasts (Liu et al., 2019). CAFs present myofibroblast-like properties, such as strong contractility and ECM deposition, and this allows CAFs to produce both chemical and mechanical signals that support invasive tumor growth (DiPersio and Van De Water, 2019). A classification system (based on immunohistochemical staining of the filaments) for myofibroblasts identified 5 distinct types, each expressing various combinations of desmin, α-SMA, vimentin, and myosin (Powell et al., 1999; Bagalad et al., 2017). All of these are also markers for smooth muscle differentiation (Wong and Tam, 2002), which occurs when tumor invades the muscle surrounding the origin organ (bladder, prostate, colon, etc.).

CAF function is regulated by integrins, which are expressed on all tumor and stromal cell types, regulating cell adhesion and bidirectional mechanotransduction across the cell membrane (DiPersio and Van De Water, 2019). Integrins regulate the capacity of CAFs to produce and respond to extracellular cues in the tumor microenvironment (DiPersio and Van De Water, 2019). Integrins can control pro-tumorigenic cell-autonomous functions within both tumor cells and stromal cells (CAFs or myofibroblasts) that promote cancer, including survival, proliferation, motility/invasion, and ECM modulation (Brakebusch et al., 2002; Missan and DiPersio, 2012; DiPersio and Van De Water, 2019). Integrins (α3β1 in work by DiPersio & Van De Water) also play a role in the regulation of intercellular communication within the TME, via regulation of paracrine signaling, either chemical or mechanical, between tumor cells and stromal cells (Brücher and Jamall, 2014; Longmate and DiPersio, 2017; DiPersio and Van De Water, 2019). In pancreatic cancer, α6β1 integrin expressed by tumor cells interacts with uPAR expressed on stromal fibroblasts and promotes activation in the fibroblasts of the uPAR–uPA–MMP-2 proteolytic cascade, aiding in tumor progression (He et al., 2007).

Integrin α11β1, a collagen adhesion receptor, plays a role in differentiation of fibroblasts into CAFs, and also is expressed by CAFs (Zeltz et al., 2019). Integrin α11β1 has been shown to promote tumor growth and metastatic potential of non-small cell lung cancer (NSCLC) cells (Navab et al., 2016), breast cancer, in conjunction with platelet-derived growth factor receptor β (PDGFRβ) (Primac et al., 2019), and in head and neck squamous cell carcinoma (HNSCC) and pancreatic ductal adenocarcinoma (PDAC) (Zeltz et al., 2019). Integrin α11β1 is highly expressed on fibroblasts, and α11β1 knockouts produce considerably reduced smooth muscle cell α-actin (SMC-actin) expression and lower tensile strength of healed wounds [reviewed in (DiPersio and Van De Water, 2019)]. Integrin α11β1 appears to be regulated by ECM stress, acting as a mechanosensor, involving activin A and Smad3. and regulates myofibroblast differentiation (Carracedo et al., 2010). It is known that α11β1 integrin binds smooth muscle cells to the ECM, so its down-regulation in tumor progression may make smooth muscle less resistant to invading tumors, as is also true in the down-regulation of α7β1 in smooth muscle when tumor invades (Tan et al., 2013).

Contrasted with normal fibroblasts, CAFs produce a fibronectin-rich ECM with anisotropic fiber orientation, which guides directional migration of cancer cells (Erdogan et al., 2017a). CAF-induced directional migration, in both the prostate cell line DU145 and the head and neck squamous cell carcinoma lines JHU012 (laryngeal squamous cell carcinoma) and SCC61 (tongue squamous cell carcinoma), suggests a non-organ-specific mechanism by which CAFs modulate cancer cell migration (Erdogan et al., 2017a). Matrix configuration by CAFs is facilitated by enhanced myosin-II–driven contractility and increased traction forces, transferred to the ECM via α5β1 integrin (Erdogan et al., 2017a). Integrin α5β1 is also produced by CAFs (Jang et al., 2019), and one of its roles is mechanosensing of the stiffness of normal tissue (Elosegui-Artola et al., 2014), but not in tumor (Jang et al., 2019). Cancer cells have been shown to adhere to fibroblasts through integrin α5β1-mediated binding to fibronectin on the surface of fibroblasts (Miyazaki et al., 2019).

DiPersio & Van De Water report that α3β1, a laminin-binding integrin, and α5β1, a fibronectin-binding integrin, are both required on CAFs for protease- and force-mediated ECM remodeling that creates matrix tracks to guide collective invasion of carcinoma cells, likely with separate signaling pathways (DiPersio and Van De Water, 2019). The authors also report research by Cavaco et al., that revealed a crucial role for α3β1 integrin, and its interaction with laminin-332, in CAF differentiation and maintenance (Cavaco et al., 2018; DiPersio and Van De Water, 2019), as well as work from their lab, and others, that revealed a central role for α3β1 integrin in epithelial cells to regulate paracrine stimulation of stromal cells (DiPersio and Van De Water, 2019). Cavaco, working in pancreatic cancer, found that α3 integrin is considerably up-regulated during tumor differentiation with much greater expression in inflammatory CAFs (iCAFs) than in the immortalized human pancreatic fibroblasts, concluding that α3β1 integrin is a marker for CAF differentiation when combined with the expression of its ligand, laminin-332 (Cavaco et al., 2018).

Erdogan et al., in working with the DU145 prostate cancer cell line, found that immunofluorescence (IF) staining of DU145-CAF co-cultures for N-cadherin and E-cadherin revealed DU145 cells forming E-cadherin junctions with other DU145 cancer cells, but the CAFs formed N-cadherin junctions with other CAFs—they did not observe any N-cadherin/E-cadherin connections at sites where DU145 cancer cells made contact with CAFs (Erdogan et al., 2017b). Labernadie et al., found that CAFs drive *in vitro* collective tumor invasion via an intercellular physical force that is transmitted through heterophilic AJs involving E-cadherin on the cancer cell membrane and N-cadherin on the CAF membrane (Labernadie et al., 2017). This heterotypic adhesion between both cell types facilitates force transmission and mechanotransduction as well as CAF polarization (Labernadie et al., 2017). Both studies show that E-Cadherin expression is maintained in collective tumor migration, but that N-Cadherin plays a crucial role as well, confirming previous data from our own research group (Tran et al., 1999b). We note with interest that exosomes have been proposed as a mechanism for altering cellular adhesion membrane components (Krishn et al., 2019; Li et al., 2022).

CAFs are the predominant type of stromal cells in the TME (Erdogan et al., 2017a; Goulet et al., 2019; Li et al., 2021), and as such, their role providing paracrine factors or exosomes in tumor initiation, growth, and metastasis has become the focus of intense research over the last 10 years. Among the many cancers being investigated, muscle-invasive bladder cancer (Goulet et al., 2019; Zhou et al., 2020), invasive colorectal cancer (Tommelein et al., 2015; Son et al., 2019), and metastatic prostate cancer (Ippolito et al., 2019; Bonollo et al., 2020) all show a central role for CAFs in the development and progression of the disease. As shown above, CAFs contribute a variety of growth factors to the TME, mediated in part by integrins, and facilitated by cadherins. CAFs are the proposed initiators of EMT in bladder cancer (Goulet et al., 2019) and colorectal cancer (Asif et al., 2021), and this extends to many other cancers, as well (Fiori et al., 2019; Mezawa and Orimo, 2021). Taken together, this data suggests a fundamental role for CAFs in the epithelial-mesenchymal cooperation we propose. This cooperative phenotype better represents the *in vivo* process than does traditional EMT conceptualizations. Future research may provide actionable targets to inhibit CAF formation in a tumor-suppressive manner, thereby reducing metastatic risk.

### 3.2 The Biophysical Properties of the Muscle

Smooth muscle invasion is a key means of tumor escape from the primary site in cancers of the prostate, bladder, esophagus, stomach, and colon/rectum (Society AC, 2020). When these tumors remain organ-confined, they are not lethal with proper treatment; however, lethality increases substantially once they escape the primary organ and metastasize. The smooth muscle is a unique biophysical tumor microenvironment, and together with connective tissue, forms the anatomical layers of submucosa, mucosa, and serosa surrounding hollow organs or organ systems (Beunk et al., 2019). Smooth muscle is characterized as involuntary, non-striated muscle, functionally distinct from skeletal and cardiac muscle, and is found in the walls of the urinary bladder, uterus, stomach, intestines, prostate, arteries and veins of the circulatory system, and in the respiratory, urinary, and reproductive systems (Betts et al., 2013; Hafen and Burns, 2020). Fully differentiated smooth muscle expresses the cytoplasmic molecular markers desmin (an intermediate filament), calponin (an actin-binding protein), and laminin-binding adhesion receptors, including the biosensing α7β1 integrin (Burkin and Kaufman, 1999; Ayala et al., 2003; Boppart et al., 2006; De Vivar et al., 2017). Compared to striated muscle, smooth muscle expresses greater elasticity and function within a larger length-tension curve, and the ability to stretch without losing contractility is necessary in organs such as the bladder, stomach, and intestines (Betts et al., 2013).

Tumors escaping their site-of-origin generally face a barrier of single-unit smooth muscle cells rather than the multi-unit type. Smooth muscle composed of single-unit muscle fibers are joined by gap junctions, which permits the whole muscle to contract as a single unit (visceral muscle) and is found in the walls of all visceral organs other than the heart (which has unique cardiac muscle in its walls) (Betts et al., 2013). Not being hindered by the limited elasticity of sarcomeres, single-unit fibers of the visceral muscle possess a stress-relaxation response that is obvious in the muscles of hollow organs such as the colon or bladder, that stretch and expand as they fill, creating a mechanical stress that generates contraction followed by relaxation in order to prevent an unfortunate release of the organ’s contents (Harryman et al., 2021). Visceral smooth muscle generally produces slow, uniform contractions that allow organ contents, such as urine, to accumulate and be released—and to be clear, striated muscle forms the urethral sphincter, allowing for voluntary release of urine (Jung et al., 2012).

Smooth muscle cells (myocytes) are found beneath the basement membrane and are constrained by loose fibrillar networks rich in collagen (Beunk et al., 2019). This structure provides tissue cohesion and plasticity of tissue movement during contraction, which allows for comparatively low resistance for migrating cells, and the basement membranes offer a variety of ligands, including laminin, for cell adhesion (Beunk et al., 2019). Invading tumor cells face a unique microenvironment when invading smooth muscle with biophysical properties such as muscular elasticity, contraction/relaxation forces, and tissue rigidity that present physical cues shaping the dynamics of tumor invasion (Beunk et al., 2019). As the tumor penetrates the muscle barrier, these biophysical forces are apt to fluctuate due to the elasticity of the muscle, which can be as much as 100 times stiffer than the layer of fat surrounding the prostate capsule and the bladder (Cox and Erler, 2011a).

Epithelial tumors employ integrins, biophysical and biochemical sensing receptors, to interact with the smooth muscle ECM (such as laminin, fibronectin, and collagen). Integrins are ECM adhesion receptors that orchestrate cell mechanics and transmission forces in the variable ECM environments during the normal processes of embryonic development and morphogenesis and in clinical developments such as epithelial tumor growth during invasion and metastasis (Goodwin et al., 2016; Mui et al., 2016; Changede and Sheetz, 2017; Harryman et al., 2021). In smooth muscle, the α7β1 integrin, which is specific for laminin binding to the ECM, is highly expressed; inactivating mutations of this integrin will cause loss of muscle integrity (Burkin and Kaufman, 1999; Boppart et al., 2006; Liu et al., 2009; Okada et al., 2013; Harryman et al., 2021). The function of α7β1 integrin is to facilitate muscle cell interactions with laminin in the ECM, while the role of α6β1 integrin (also specific for laminin) is to mediate tumor cell–ECM interactions and smooth muscle invasion (Rubenstein et al., 2019; Harryman et al., 2021), suggesting that there may be integrin cooperation between the invading tumor and the normal muscle during laminin-based invasion (Harryman et al., 2021). There is still little known about the various elements that promote tumor migration from the primary organ into and through the smooth muscle barrier, and there likely will be a combination of biomechanical forces, chemoattractants, biochemical signals, CAFs, and hormonal factors that reduce resistance in the muscle to the invasive tumor (Harryman et al., 2021).

## 4 The Growth Factors That Influence Tumor Integrin Function in the Muscle

**FIGURE 2:**
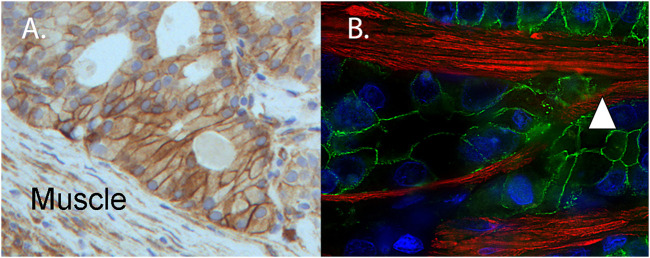
Muscle invasive patient-derived xenograft (PDX) expresses kindlin-2 and angulated invasive clusters of human tumor (from a surgical specimen) splitting the muscle. **(A)** Immunohistochemistry of PDX MDA PCa 173-2-4 shows expression of kindlin-2 (brown, DAB) on tumor cell surface of angulated cluster invading mouse muscle (Muscle). **(B)** For comparative purposes, a radical prostatectomy specimen contains angulated invasive human prostate cancer expressing E-cadherin (green), splitting the muscle (white triangle) (desmin, red). Nuclei detected by DAPI (DNA, blue).

A major transition in the progression of prostate and bladder cancer to lethal disease is the ability of the tumor to invade into and through organ-confining muscle layers. In prostate cancer, the structural integrity of the muscle layer of the prostate capsule becomes progressively disrupted. Microscopically, the muscle becomes disorganized in the presence of the activated tumor, as inferred by the muscle response and the observation that tumor clusters within the muscle become highly angulated (Ayala et al., 2003). An essential regulator of morphogenesis of the epithelium and cancer invasion and metastasis is kindlin-2 (Wang et al., 2020; Zhu et al., 2021). Our research group has observed an increased expression of kindlin-2, an integrin adapter, indicating integrin activation within a patient-derived xenograft model (Palanisamy et al., 2020). The cell surfaces of the angulated tumor cluster express kindlin-2 (Figure 2A). Angulated tumor within a radical prostatectomy specimen (Figure 2B) shows the presence of E-cadherin in a cell-cell distribution within the tumor cell clusters as the muscle is parted. We speculate that kindlin 2 may be an important molecular regulator of the tumor invasive networks to move through muscle. Recent work has highlighted emerging evidence for kindlin oligomerization and integrin clustering as mechanisms to regulate integrin function (Bu et al., 2021; Soe et al., 2021).

Early work recognized the importance of reciprocal interactions of the tumor and muscle that coordinatively resulted in organ escape (Ayala et al., 2003). While matrix metalloproteinases (MMPs) and corresponding ECM changes occur during prostate cancer progression (Harryman et al., 2019), it is unknown how the muscle and tumor alterations cooperate. Recent work has centered on the view that the plasticity of cell phenotypes in both compartments may be responsive to growth factors and secreted proteins as agents of change.

Maintenance of the differentiated phenotype of smooth muscle occurs using the IGF-1 signaling pathway through PI3-kinase (Hayashi et al., 1998). Insulin-like growth factors (IGF-I and IGF-II) and insulin markedly prolonged the differentiated phenotype with IGF-I being the more potent. The secretion of IGF-I is enhanced by the extracellular matrix protein, laminin. In contrast, serum epidermal growth factor (EGF) and platelet derived growth factors (PDGF) can induce de-differentiation as well as being potent inhibitors of the downstream pathway of PI3 kinase (wortmannin and LY294002). The inhibitors induced de-differentiation of the muscle even in the face of IGF-I stimulation (Hayashi et al., 1998). Further work showed that the balance of PI3-kinase/AKT and ERK/p38MAPK will change the differentiation status of muscle (Hayashi et al., 1999). In another study, using primary cultured vascular smooth muscle cells, laminin induced a delay in the progression of de-differentiation, maintaining the contractile phenotype, whereas fibronectin stimulated it (Hedin et al., 1988). The results suggested diverse functional roles of fibronectin and laminin as ECM molecules in control of the differentiated properties of smooth muscle. Taken together, these data suggest that contextual signals provided by ECM proteins can remarkably affect the action of growth factors to either maintain differentiation or induce a de-differentiation response in adult smooth muscle.

One mechanism for PDGF to induce de-differentiation of smooth muscle, via downstream kinase signaling pathways of ERK and JNK, is mediated through a secreted cellular communication network factor 1 protein (CCN1; CYR61 gene) (Zhang et al., 2015). CCN1 also is associated in several epithelial cancers with a pro-metastatic phenotype that can be blocked to prevent metastasis (Ilhan et al., 2020) and with a drug resistance phenotype (Vidot et al., 2010). The role of extracellular CCN1 to alter both the de-differentiation of muscle and stimulation of tumor aggressive phenotypes may be related to its ability to directly interact with the laminin-binding integrin class of cell adhesion receptors. CCN1 interacts with both endothelial and epithelial specific α6β1 integrin and α7β1 integrin, another laminin-binding integrin found on muscle cells (Leu et al., 2003). We hypothesize that CCN1 provides the contextual signal to modulate both the epithelial and muscle laminin-binding integrins. We further speculate that the reported paradoxical effects of CCN1 to alter phenotypes (Lu and Kang, 2016; Park et al., 2019; Oliphant et al., 2020) is both cell-type and context dependent for autocrine and paracrine effects. While the current literature supports the idea that CCN1 is a biologically active paracrine factor for tissue remodeling, we propose that CCN1 could act as a key modulator mediating the cross talk between epithelial tumor migration into and through the muscle, and the known muscle remodeling that occurs during tumor progression. We note with interest that current tumor transcriptional databases cannot distinguish whether CCN1 expression changes are due to CCN1 expressed by the tumor, muscle, or endothelium. Understanding the functional relevance of increased CCN1 associated with increasing grade or worse patient outcomes is difficult when the source of CCN1 is not known in these complex samples. This is especially important since muscle loss in response to a resident tumor is a known progression event that is manifested as reduced desmin and smooth muscle alpha-actin as hallmarks of cancer-associated reactive stroma relative to normal fibromuscular stroma (Ayala et al., 2003). Future work to spatially profile expression levels of protein and mRNA in different cellular compartments in the context of the smooth muscle microenvironment will be important to increase our understanding.

Muscle, as a microenvironment for cancer, is a contractile and stiff environment as compared to interstitial stroma, endothelium, or fat (Cox and Erler, 2011b). Matrix stiffness in tumor model systems can induce endothelial CCN1 secretion and was postulated as an autocrine factor in the endothelium and functioning in a paracrine fashion as a tumor cell adhesion ligand for gaining access to the circulation (Reid et al., 2017; Reid and Zanivan, 2017). In a similar manner, the contractile smooth muscle microenvironment may induce CCN1 secretion as a de-differentiation factor in the muscle and function as a paracrine factor to clear a path for tumor invasion into and through the smooth muscle environment.

The secretion of cytokines (IL6, TNF-alpha) can be stimulated to induce de-differentiation of muscle cells. For example, bacterial lipopolysaccharide (LPS) can change the muscle differentiation phenotype within 24 h of exposure (Leimgruber et al., 2011). The resulting secretory phenotype was confirmed by electron microscopy, immunofluorescence, and direct loss of calponin with an increase in vimentin levels in muscle cells. Similarly, the influx of macrophages and inflammatory cytokines are required for dual beneficial roles of proliferation and differentiation to accomplish muscle regeneration (Chazaud et al., 2009). While several triggers and growth factors are required, the resulting plasticity of the smooth muscle is heritable and can be local in effect.

Smooth muscle plasticity is regulated epigenetically via histone modification, DNA methylation, and demethylation triggered by different growth factors to influence their phenotypic state (Liu et al., 2015). Smooth muscle cell (SMC) dedifferentiation occurs by stabilizing DNA methyltransferase 3A (DNMT3A). Reduced DNMT3A protein leads to DNA hypomethylation in contractile gene promoters, which increased SMC contractile protein expression. DNMT3A degradation via E3 ligase TRAF6 drives differentiation of SMCs (Jeong et al., 2021). A unifying epigenetic mechanism also has been proposed that confers reversible SMC differentiation governed by the DNA-modifying enzyme ten-eleven translocation-2 (TET2). Loss of TET2 and 5-hmC positively correlates with the degree of injury in murine models of smooth muscle injury. Importantly, localized TET2 knockdown exacerbates injury response, and local TET2 overexpression restores the 5-hmC epigenetic landscape and contractile gene expression. TET2 is viewed as a novel and necessary master epigenetic regulator of SMC differentiation (Liu et al., 2013). Further studies identify the H3K4me2-TET2-miR145 axis as a central epigenetic memory mechanism controlling muscle cell identity and function (Liu et al., 2021). Taken together these data suggest that the smooth muscle phenotypic plasticity is a regulated event by growth factors that could explain the disruption of the muscle during an injury event such as tumor invasion. The potential reversibility of the de-differentiation response of the muscle to the tumor will be an interesting avenue of future study.

## 5 Conclusion

The evolving EMT paradigm includes a recent shift in understanding that invasive and metastatic tumor cell clusters contain phenotypic diversity and heterogeneity. Tumor cell phenotypic heterogeneity is a predominant feature within clusters of metastatic tumor cells, directly observable by the multiplex digital spatial profiling of proteins and RNA in fixed tissue (Merritt et al., 2020). Epithelial-mesenchymal cooperation (EMC) is dynamic and responsive to changing environmental conditions, resulting in adaptive resistance, a challenge for optimal therapeutic intervention (Labrie et al., 2019). Transient commensal clonal interactions of tumor can drive metastasis (Naffar-Abu Amara et al., 2020), with significant functional heterogeneity (Kuiken et al., 2021).

The invasion of tumor clusters into smooth muscle represents an early event in the metastatic cascade. Work is just beginning to understand the regulation of tumor cluster invasion into and through the smooth muscle for systemic spread. Integrins and growth factors, modulated by CAFs, dramatically influence epithelial-mesenchymal cooperation within the tumor clusters for invasion into and through the muscle. In addition, the mechanosensing features of the integrins and cell-cell strength properties of E-cadherin likely cooperate for tumor survival within the contractile nature of the smooth muscle. The use of patient-derived xenograft models will increase our understanding of the phenotypic functional heterogeneity in early tumor invasive networks to aid identification of new tumor subtypes responsible for an aggressive transition. The combined use of digital spatial profiling of tumor and the smooth muscle microenvironment coupled with non-invasive imaging techniques holds great promise for early detection of the initial stages of metastasis, especially for prostate and bladder cancer patients, to aid therapeutic decisions. It is currently unknown how the alterations of cell adhesion receptors are coordinated during epithelial mesenchymal cooperation. Future work on digital spatial profiling of tumors within the complex environment of muscle invasion is likely to be very informative for understanding cooperative heterogeneity. We also note the recent advances in multi-parametric MRI imaging to non-invasively observe extraprostatic extension in prostate cancer and the potential to combine this with PET imaging for early detection and prevention of the earliest stage of metastasis (Guglielmo et al., 2021; Skawran et al., 2022).

## Data Availability

Original image contributions are presented in the study and further inquiries can be directed to the corresponding author.
